# Independent Recruitment of a Flavin-Dependent Monooxygenase for Safe Accumulation of Sequestered Pyrrolizidine Alkaloids in Grasshoppers and Moths

**DOI:** 10.1371/journal.pone.0031796

**Published:** 2012-02-20

**Authors:** Linzhu Wang, Till Beuerle, James Timbilla, Dietrich Ober

**Affiliations:** 1 Biochemical Ecology and Molecular Evolution, Botanical Institute and Botanical Garden, Christian-Albrechts-Universität, Kiel, Germany; 2 Institute for Pharmaceutical Biology, Technical University Braunschweig, Braunschweig, Germany; 3 Queensborough Community College, City University of New York, New York, New York, United States of America; Universidade de São Paulo, Brazil

## Abstract

Several insect lineages have developed diverse strategies to sequester toxic pyrrolizidine alkaloids from food-plants for their own defense. Here, we show that in two highly divergent insect taxa, the hemimetabolous grasshoppers and the holometabolous butterflies, an almost identical strategy evolved independently for safe accumulation of pyrrolizidine alkaloids. This strategy involves a pyrrolizidine alkaloid *N*-oxygenase that transfers the pyrrolizidine alkaloids to their respective *N*-oxide, enabling the insects to avoid high concentrations of toxic pyrrolizidine alkaloids in the hemolymph. We have identified a pyrrolizidine alkaloid *N*-oxygenase, which is a flavin-dependent monooxygenase, of the grasshopper *Zonocerus variegatus*. After heterologous expression in *E. coli*, this enzyme shows high specificity for pyrrolizidine alkaloids of various structural types and for the tropane alkaloid atropine as substrates, a property that has been described previously for a pyrrolizidine alkaloid *N*-oxygenase of the arctiid moth *Grammia geneura*. Phylogenetic analyses of insect flavin-dependent monooxygenase sequences suggest that independent gene duplication events preceded the establishment of this specific enzyme in the lineages of the grasshoppers and of arctiid moths. Two further flavin-dependent monooxygenase sequences have been identified from *Z. variegatus* sharing amino acid identities of approximately 78% to the pyrrolizidine alkaloid *N*-oxygenase. After heterologous expression, both enzymes are also able to catalyze the *N*-oxygenation of pyrrolizidine alkaloids, albeit with a 400-fold lower specific activity. With respect to the high sequence identity between the three *Z. variegatus* sequences this ability to *N*-oxygenize pyrrolizidine alkaloids is interpreted as a relict of a former bifunctional ancestor gene of which one of the gene copies optimized this activity for the specific adaptation to pyrrolizidine alkaloid containing food plants.

## Introduction

**Figure 1:**
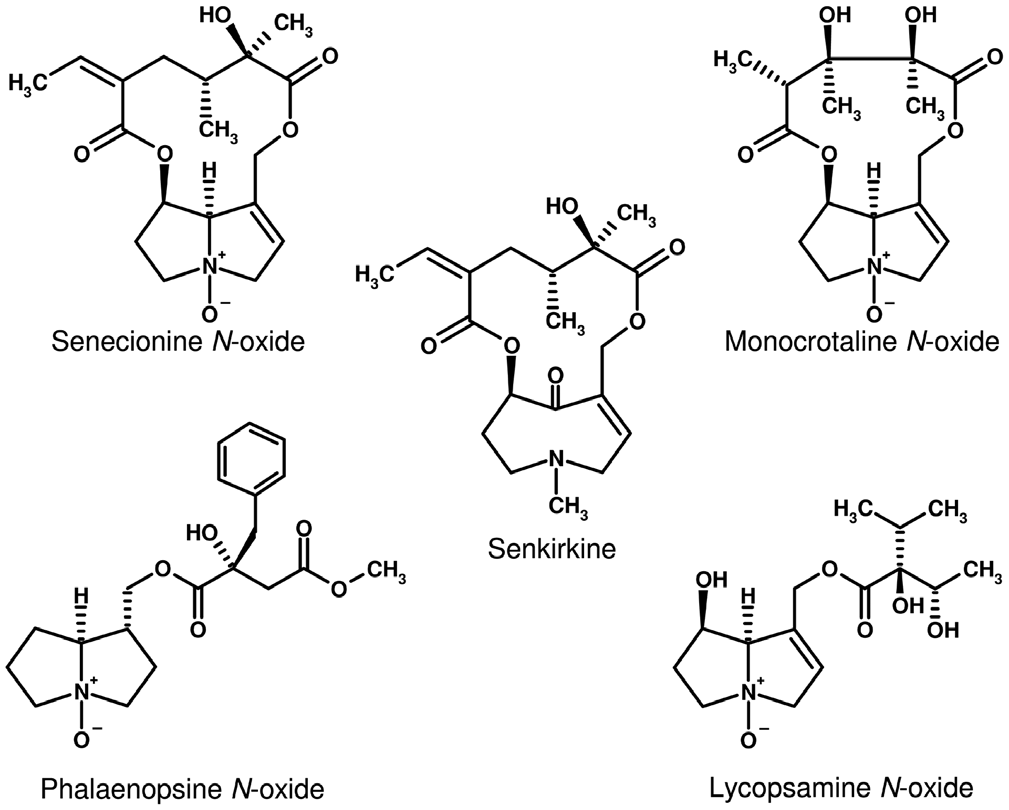
Structures of characteristic pyrrolizidine alkaloids. Structures are given in the *N*-oxide form with the exception of the otonecine derivative, senkirkine.

Chemical defense against herbivory is essential for plants to be able to survive in their natural habitat. During evolution, many insect herbivores have developed counterstrategies to cope with these toxic compounds. In some cases, they have even acquired these chemicals for their own benefit. One of the best studied examples of plant toxins sequestered by adapted insects are the pyrrolizidine alkaloids (PAs) that are found in certain lineages scattered within the angiosperms [1]. PAs occur in plants usually in their polar non-toxic *N*-oxide form (Figure 1). After ingestion by a vertebrate or insect herbivore, the *N*-oxides are easily reduced to the protoxic free base, the substrate for cytochrome P450-mediated bioactivation [2], [3].

Strategies for PA sequestration in adapted insects have evolved in various insect lineages under the identical selection pressure to avoid higher concentrations of PAs in the form of their free base in the hemolymph (for recent reviews see [1], [3], [4], [5]). Leaf beetles of the genus *Platyphora* (Chrysomelidae, Coleoptera) have developed a strategy to transfer the free base from the hemolymph into defense secretions with such an efficiency that all other tissues outside the secretory glands are almost devoid of PAs [6]. In the related leaf beetle genus *Oreina* (Chrysomelidae, Coleoptera), the reduction of ingested PAs is suppressed. Instead, the *N*-oxides are directly absorbed and accumulated in their hemolymph and defense glands [7], [8]. A third strategy to handle sequestered PAs is the stabilization of the PAs by enzyme-catalyzed *N*-oxidation of the alkaloids within the insect. This mechanism is realized in larvae of the tiger moth family (Arctiidae, Lepidoptera) [9]–[13], in several *Longitarsus* flea beetle species [14], [15], and in the grasshopper genus *Zonocerus* [13], [16]. Recently, we have been able to show that the enzyme responsible for the *N*-oxygenation of PAs in arctiids belongs to the family of flavin-dependent monooxygenases (FMOs) [12], [13] and was recruited by gene duplication early in this lineage during the adaptation to PA-containing plants [17]. To date, these PA *N*-oxygenases are the only functional characterized FMOs of insects.

FMOs are well characterized in vertebrates, where they are involved in the detoxification of nucleophilic nitrogen- and sulfur-containing xenobiotics [18]. Although insects have to cope with a wide variety of xenobiotics, none of these so-called microsomal multisubstrate FMOs has been detected in insects, suggesting that the xenobiotic metabolism is guaranteed by cytochrome P450 monooxygenases (CYPs). Despite the different mechanism by which these two classes of monooxygenases operate, both convert lipophilic compounds into more hydrophilic metabolites that are readily excreted and have reduced bioactivity [3], [18]. Both, FMOs and CYPs have been shown to catalyze the *N*-oxygenation of PAs in vertebrates [19], [20].

Here, we report that PA *N*-oxygenation as one strategy the for insect adaptation to PA-containing food plants evolved independently in the grasshopper *Z. variegatus* and the arctiid moths. We show that convergent evolution resulted in two almost identical systems. This identity refers to the findings that, in both cases, an FMO has been recruited, and that the respective enzymes show almost identical substrate specificity.

## Materials and Methods

### Collection and rearing of *Zonocerus*

Larvae and adults of *Zonocerus variegatus* were collected in the vicinity of Accra, Ghana, and reared in the laboratory under a light/dark regime of 16/8 h at 20 to 25°C. Egg pots were incubated in moist sand at a constant temperature of 30°C. Nymphs and adults were fed with leaves of *Rubus fruticosus* unless otherwise stated. Leaves of *R. fruticosus* are regarded as non-toxic as they do not contain PAs.

### RNA isolation and cDNA synthesis

Adult insects were dissected, and fat body tissue was frozen in liquid nitrogen before total RNA was isolated by using TRIZOL® reagent (Invitrogen); 2 µg RNA was used for cDNA synthesis with Superscript III reverse transcriptase (Invitrogen) and an oligo(dT)_17_ primer (P1, 0.1 µM; Table S1) at 55°C in a total volume of 20 µl.

### Identification and expression of FMO-like cDNA sequences

Degenerate primers P2 and P3 (for primer sequences see Table S1) were designed based on an alignment of FMO sequences of the lepidopteran species *Arctia caja*, *A. villica*, *Tyria jacobaeae*, and *Grammia geneura* (Syn.: *G. incorrupta*) in combination with two FMO-like sequences identified in each of the genomes of *Anopheles gambiae* and *Drosophila melanogaster*. In a semi-nested PCR approach, 3 µl of *Z. variegatus* cDNA was amplified with AccuTaq LA polymerase (Sigma) and primer pair P2/P1 by using a touch-down protocol with decreasing annealing temperatures from 60°C to 52°C (−0.5°C per cycle for 16 cycles and 52°C constant for 20 further cycles). The reaction was diluted 1∶100, and 3 µl was used as template for the second PCR with primer pair P2/P3 and a decreasing annealing temperature from 55°C to 47°C in a total volume of 25 µl. The resulting fragment of 473 bp was subcloned by using the pGEM-T Easy vector (Promega). Sequencing revealed similarity to the FMO-encoding sequences of insects. Gene-specific primers were designed and used for 3′- and 5′-rapid amplification of cDNA ends (RACE, P4–P7) as described previously [21]. 3′-RACE resulted in the identification of two sequences covering the 3′-cDNA end and sharing 86% sequence identity. One of these sequences overlapped with the fragment identified with the degenerate primers and with the 5′-RACE fragment and was assembled to the full-length sequence (*Zv*FMOa). According to the second 3′-end cDNA, gene-specific primers (P8–P10) were designed that were used for identification by 5′-RACE. The resulting second full-length sequence was named *Zv*FMOc. For the amplification of the full open reading frames (ORFs) of *Zv*FMOa and *Zv*FMOc, primer pairs P11/P12 and P13/P14 were used, respectively, at an annealing temperature of 55°C with Platinum *pfx* DNA polymerase (Invitrogen), which possesses proof-reading activity. The resulting fragments were digested with NdeI/XhoI and cloned into an NdeI/XhoI-linearized pET22b vector for heterologous expression with a C-terminal hexahistidine tag in *E. coli* BL21(DE3). Restriction of the fragment resulting from amplification with primer pair P13/P14 (*Zv*FMOc) indicated that the PCR product was not homogeneous, as a fragment was detected that contained an internal, non-predicted restriction site for XhoI. Cloning of this fragment into the pGEM-T easy vector, screening with XhoI, and sequencing resulted in the identification of another FMO-encoding sequence, termed *Zv*PNO. For this sequence, the 3′- and 5′-cDNA ends were identified by RACE with the primers P15–P19. The full ORF of *Zv*PNO was amplified with primer pair P20/P21 at 55°C with Platinum *pfx* DNA polymerase, digested with BsaI and NotI, and cloned into an NcoI/NotI-linearized pET28a vector for expression with a C-terminal hexahistidine tag in *E. coli* BL21(DE3). Expression of the recombinant proteins *Zv*FMOa, *Zv*FMOc, and *Zv*PNO was induced with 0.1 mM isopropyl β-D-thiogalactoside at 30°C, before the proteins were purified by metal chelate affinity chromatography by using Ni^2+^-nitrilotriacetic acid-agarose (Qiagen). For determination of the native *M*_r_, the purified enzyme was applied to a Superdex200HighLoad column (GE Healthcare) using 20 mM glycine/NaOH buffer pH 9.0 containing 200 mM NaCl. As reference proteins thyroglobulin (669 kDa), ferritin (440 kDa), bovine serum albumin (67 kDa), ovalbumin (43 kDa), and chymotrypsinogen A (25 kDa) have been used.

### Sequence analysis

Comparison of amino acid sequences was performed with the Bestfit software of the Wisconsin Sequence Analysis Package (version10, Genetics Computer Group, Madison, WI). An alignment of amino acid sequences (Figure S1) was generated with ClustalX [22] and used to estimate phylogenies with the PHYLIP program package [23]. The maximum likelihood tree was calculated with PROML by using the Jones-Taylor-Thornton model for amino acid changes [24]. Bootstrap values were estimated with the programs SEQBOOT and CONSENSE. The bootstrap estimates are the result of 1000 replicates. The sequences reported in this paper have been submitted to the EMBL Nucleotide Sequence Database with the accession numbers FR696371 (*Zv*FMOa), FR696372 (*Zv*FMOc), and FR696373 (*Zv*PNO).

### Assay for PA *N*-oxygenase activity

Enzyme activity was assayed in a 100 mM glycine/NaOH buffer pH 9 containing 0.2 mM NADPH at 37°C photometrically or in a radioassay as described previously [12]. All substrates were assayed at a concentration of 0.2 mM by using the photometric assay or the radioassay (0.023 µCi/assay), except for retronecine, supinidine, and isoretronecanol, which were tested as ^3^H-labeled substrates, and phalaenopsine as a ^14^C-labeled substrate in the radioassay (0.023 and 0.045 µCi/assay, respectively). For enzyme kinetics, initial rates were determined by varying the concentration of alkaloid (1–40 µM for senecionine, 4–60 µM for monocrotaline and atropine, and 0.1–1.5 mM for heliotrine and phalaenopsine) while maintaining the concentration of NADPH at 200 µM. The identity of senecionine *N*-oxide, seneciphylline *N*-oxide, monocrotaline *N*-oxide, phalaenopsine *N*-oxide, and atropine *N*-oxide was confirmed by liquid chromatography mass spectrometry (LC-MS). For LC-MS analysis, samples were diluted by using 50% acetonitrile containing 1% formic acid. Solutions were infused directly into a 3200 QTrap MS instrument (Applied Biosystems/MDS Sciex). The mass spectrometer was equipped with an electrospray ionization interface (ESI, Turbo V) and was operated in positive-ion mode and enhanced product ion (EPI) scan mode. Ionization and EPI conditions were optimized individually for each compound in its non-oxidated form, and the obtained settings were used to analyze extracts of the corresponding enzymatic assays. Nitrogen was used as a curtain and auxiliary gas.

## Results

### Identification and Heterologous Expression of PA *N*-Oxygenase of *Z. variegatus*

In contrast to the arctiid moths in which the PA *N*-oxygenase is a soluble protein in the hemolymph, the alkaloid *N*-oxygenating activity in the grasshopper *Z. variegatus* is associated with the fat body [13]. Separate incubation of a particulate fraction and the supernatant obtained from the insects fat body with ^14^C-seneciphylline showed that *N*-oxygenation activity is detectable in the soluble fraction, but not in the pellet. These results indicate that PNO is expressed as a soluble protein, excluding the involvement of a membrane-bound CYP. Therefore, we hypothesized that the enzyme might belong to the FMOs in this species. Using cDNA preparations of fat body tissue of *Z. variegatus*, we applied a polymerase chain reaction (PCR)-based approach, in order to identify FMO-encoding cDNA sequences. Degenerate primers were designed according to an alignment of FMO sequences of *Drosophila melanogaster* and *Anopheles gambiae* present in the database and of four arctiid species identified recently in our group, *viz.*, *Arctia caja*, *A. villica*, *Tyria jacobaeae*, and *Grammia geneura* [17]. Three cDNA sequences were identified, *Zv*FMOa, *Zv*FMOc, and *Zv*PNO, with open reading frames of 1242 bp, 1245 bp, and 1242 bp in length, encoding proteins with a subunit size of 47,726 Da, 47,939 Da, and 47,793 Da, respectively. The isoelectric point of the three proteins is predicted to be 6.6, 6.2, and 6.0, respectively. The sequence motifs most characteristic for FMO, *viz.*, the nucleotide-binding sites (Rossman folds, consensus GxGxxG) for binding of FAD as a prosthetic group and of the NADPH cofactor, respectively, and the FMO-identifying sequence (consensus FxGxxxHxxxY/F) are highly conserved in sequence position in comparison with the FMOs of other eukaryotes [25]–[28] (Figure S1). For none of the three sequences an *N*-terminal signal peptide was predicted as it was shown to be present in the SNO of *Tyria jacobaeae* and the FMOs of other arctiid species [12], [17]. No transmembrane helices were detected as they are present in mammalian FMOs close to the C-terminus for membrane attachment [29]. The FMO-like sequences of *Z. variegatus* share amino acid identities of 77% to 78% between each other and identities of 35% to 40% to the sequences encoding the senecionine *N*-oxygenase of *T. jacobaeae* (Lepidoptera), to the FMOs of *Drosophila melanogaster* (Diptera), and to the human FMO1 (Table 1).

**Table 1:** Degree of identity between amino acid sequences encoding FMOs of *Z. variegatus*, other insects, and the FMO1 of human.

(%)	*Zv*FMOa	*Zv*FMOc	*Zv*PNO	*Tj*SNO	*Dm*FMO3006	*Dm*FMO3174	*Hs*FMO1
*Zv*FMOa	100	77.0	77.8	35.2	36.4	36.3	39.8
*Zv*FMOc			77.0	35.3	38.2	36.6	38.8
*Zv*PNO				36.3	38.2	37.1	37.7

For comparison, the full coding regions were used. *Tj*SNO, Senecionine *N*-oxygenase of *Tyria jacobaeae*; *Dm*FMO3006 and *Dm*FMO3174, FMO sequences of unknown function present in the genome of *Drosophila melanogaster*; *Hs*FMO1, FMO1 of human.

### Biochemical Characterization of PA *N*-Oxygenase of *Z. variegatus*

After the expression and purification by metal chelate affinity chromatography, *Zv*FMOa, *Zv*FMOc, and *Zv*PNO showed, in assays for PA *N*-oxygenation, specific activities of 123 pkat/mg, 146 pkat/mg, and 59 nkat/mg, respectively (100 mM glycine/NaOH buffer pH 9.0 with 0.2 mM senecionine as substrate). The identity of the reaction product as senecionine *N*-oxide was confirmed by LC-MS (liquid chromatography with mass spectrometry). Therefore, *Zv*PNO was designated as PA *N*-oxygenase (PNO). Recombinant *Zv*PNO shows a pH optimum of 9.0 in potassium phosphate buffer (10 mM), Tris/HCl buffer (10 mM), and in glycine/NaOH buffer (100 mM), respectively (Figure S2). Using the potassium phosphate-based buffer system (pH9.0) in the standard assay, linear rates of product formation were maximal at a temperature of 42°C. Using a temperature range of 20–42°C, the activation energy was estimated to be 70.7 kJ/mol. The molecular mass of the heterologously expressed protein has been estimated by gel filtration chromatography to be 91,200 Da, indicating that the active enzyme is a homodimer.

### Substrate Specificity of Recombinant FMO of *Z. variegatus*

*Z. variegatus* is a generalist feeding on a wide array of food plants and encountering a huge diversity of plant allelochemicals [30]. Therefore, in testing the substrate specificity of recombinant *Zv*FMOa, *Zv*FMOc, and *Zv*PNO, we used, in addition to various structures of PAs, other alkaloids and compounds known to be accepted as substrates of mammalian and yeast FMO (Table 2). With regard to *Zv*PNO, PA structures of the five main structural types are *N*-oxygenized. The otonecin derivative senkirkine, which carries a methyl group at the ring-bound nitrogen, is not a substrate (Figure 1). Atropine, a tropane alkaloid common to certain solaneceous plants, is converted to its *N*-oxide form with almost the same activity as the best substrates tested, e.g., senecionine and seneciphylline. Of the substrates described for mammalian FMOs and for the FMO from *Saccharomyces cerevisiae*, cysteamine is accepted by the PNO of *Zonocerus*, whereas L-cysteine, dimethylaniline, hydroxylamine, or glutathione are not substrates. *Zv*FMOa and *Zv*FMOc show low but unequivocal activity with most of the substrates that show activity with *Zv*PNO.

**Table 2:** Substrate specificity of recombinant flavin-dependent monooxygenases of *Z. variegatus*.

Substrate	Specific activity (nkat/mg)			
	*Gg*PNO	*Zv*PNO	*Zv*FMOa	*Zv*FMOc
Pyrrolizidine alkaloids				
Senecionine type				
Senecionine	55.0	32.0	0.1	0.1
Seneciphylline	53.2	29.4	0.6	0.1
Senecivernine	-	24.0	1.3	0.6
Retrorsine	-	18.6	1.5	0.5
Senkirkine	n.d.	n.d.	n.d.	n.d.
Monocrotaline type				
Monocrotaline	90.2	23.4	1.5	0.9
Axillarine	62.2	18.6	n.d.	n.d.
Axillaridine	-	11.8	n.d.	n.d.
Lycopsamine type				
Heliotrine	44.2	19.5	n.d.	n.d.
Rinderine	38.8	11.5	1.3	0.4
Indicine	-	4.8	n.d.	n.d.
Triangularine type				
Sarracine	-	16.0	0.4	0.3
Phalaenopsine type				
Phalaenopsine	17.1	9.6	n.d.	n.d.
Necine bases				
Retronecine	n.d.	10.2	n.d.	0.1
Heliotridine	-	n.d.	n.d.	n.d.
Supinidine	n.d.	n.d.	n.d.	n.d.
Other alkaloids				
Ephedrine	-	n.d.	n.d.	n.d.
Nicotine	n.d.	2.3	0.3	0.4
Atropine	18.9	29.1	0.3	n.d.
Other substrates				
Dimethylaniline	n.d.	n.d.	n.d.	n.d.
Cysteamine	n.d.	7.0	0.5	0.1
L-Cysteine	n.d.	n.d.	n.d.	n.d.
Hydroxylamine	-	n.d.	n.d.	n.d.
Glutathione	37.0	n.d.	n.d.	n.d.

n.d., not detectable; -, not tested.

PA *N*-oxygenase (*Zv*PNO) and two related FMOs of *Z. variegatus* (*Zv*FMOa and *Zv*FMOc) have been assayed in comparison to the previously characterized recombinant PA *N*-oxygenase (*Gg*PNO) of *G. geneura* (Arctiidae, Lepidoptera) [17].

For *Zv*PNO, enzyme kinetics have been established by using selected substrates representing the major classes of PAs and of atropine. The apparent *K*_m_ values of 1.1 µM (senecionine), 11.9 µM (monocrotaline), 263.9 (heliotrine), 837.6 µM (phalaenopsine), and 9.8 µM (atropine) show that the PNO has a higher affinity to the macrocyclic diesters senecionine and monocrotaline than to the monoesters heliotrine and phalaenopsine. The affinity to the tropane alkaloid atropine is in the same range as that for the macrocyclic diester type. The values for the catalytic efficiency (*k*_cat_/*K*_m_) support the interpretation that senecionine is the most efficient substrate for the PNO followed by monocrotaline and atropine (Table 3).

**Table 3:** Enzyme kinetics of recombinant *Zv*PNO.

	*K*_m_ (µM)	*V*_max_ (nkat/mg)	*k*_cat_ (1/s)	*k*_cat_/*K*_m_ (s^−1^ mol^−1^ l)
senecionine	1.08±0.07	20.73±0.24	0.99	916 666
monocrotaline	11.93±0.76	19.99±0.40	0.96	80 469
heliotrine	263.89±10.06	2.49±0.03	0.12	454
phalaenopsine	837.61±77.37	2.66±0.11	0.13	155
atropine	9.83±1.04	7.68±0.23	0,37	37 640

For calculations a molecular mass of *Zv*PNO of 47793 g/mol was used.

### Comparative Sequence Analysis of Insect FMOs

**Figure 2:**
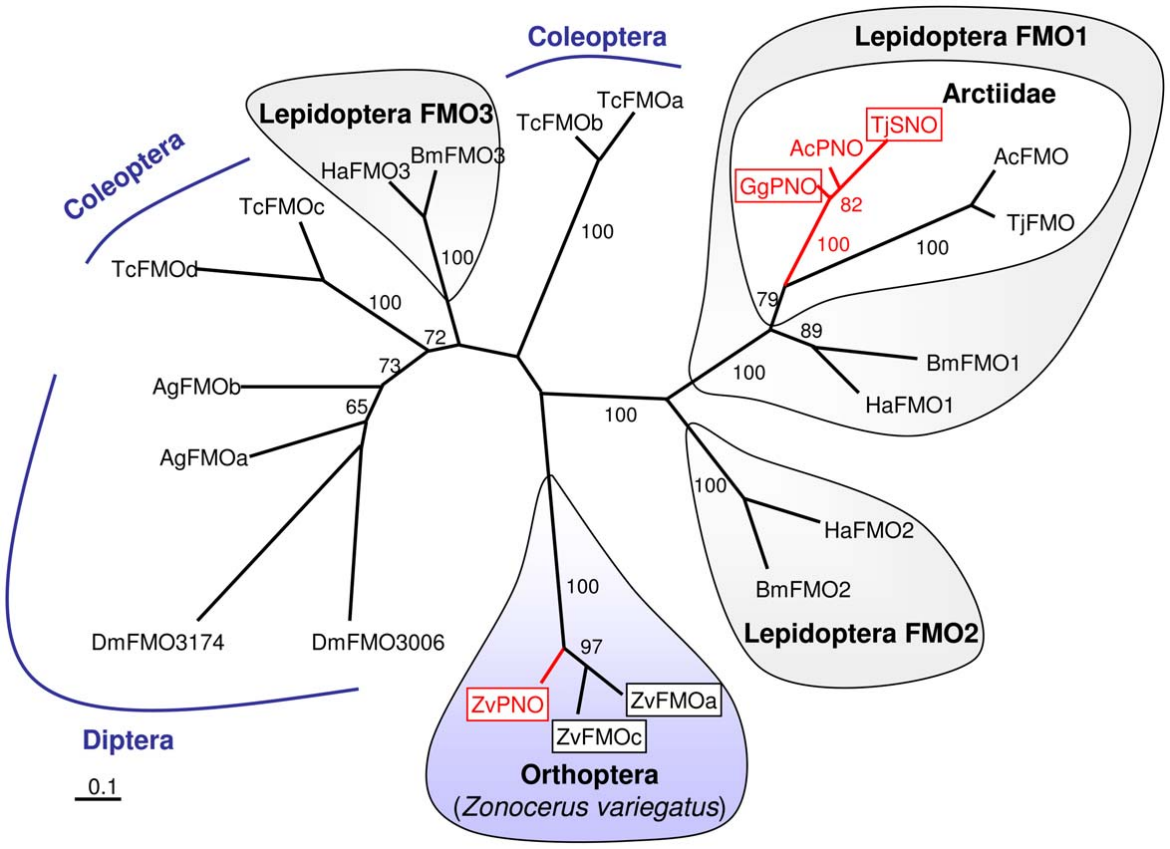
Unrooted maximum-likelihood tree of amino acid sequences derived from cDNA encoding FMOs of various insect species. Framed sequences were heterologously expressed and functionally analyzed. The other sequences should be regarded as putative FMO-coding cDNA. Branch lengths are proportional to the number of amino acid substitutions per site (scale: 0.1 substitutions per site). Bootstrap proportions resulted from 1000 replicates and are given for values >50. *Ac*, *Arctia caja*; *Ag*, *Anopheles gambiae*; *Bm*, *Bombyx mori*; *Dm*, *Drosophila melanogaster*; *Gg*, *Grammia geneura*; *Ha*, *Helicoverpa armigera*; *Tc*, *Tribolium castaneum*; *Tj*, *Tyria jacobaeae; Zv*, *Zonocerus variegatus*. Accession numbers for all sequences are listed in Figure S1.

The amino acid sequences of the three FMOs of *Z. variegatus* and of various FMOs of insects have been used for phylogenetic analysis (Figure 2). The sequences form well-supported individual clusters with regard to the sequences of the Diptera (*D. melanogaster* and *A. gambiae*) and of *Z. variegatus*, two clusters for the sequences of *Tribolium castaneum*, and three separate clusters for lepidopteran sequences, recently termed FMO1 to FMO3 [17]. Within the Lepidoptera, gene duplication events have been shown to be responsible for the separation of the related FMO1 and FMO2 cluster and, within the Arctiids, for two separated clusters, of which one encodes the PA *N*-oxygenases [17]. The tree topology clearly supports two independent origins of PA *N*-oxygenizing enzymes in insects, one early in the arctiid lineage and one in grasshoppers.

## Discussion

Our studies show that, in Arctiids and in the grasshopper *Z. variegatus*, FMOs were recruited independently as *N*-oxygenases for the safe handling of plant-derived PAs in these insects. Therefore, PA *N*-oxygenation in insects seems to be a unique feature of FMOs. Of note, CYPs in insects form a far larger gene family than FMOs (the genomes of *D. melanogaster* and *B. mori* encode 83 and 86 putative CYPs, respectively, in contrast to only 2 and 3 putative FMOs, respectively [12], [17], [31], [32]) and are well-known for their importance in the metabolism of natural and artificial xenobiotics, including insecticides [33]. Specific and inducible CYP-encoding genes in *Papilio* butterflies (Papilionidae, Lepidoptera) for the detoxification of furanocoumarins represent only one of several fascinating examples of gene evolution during adaptation of insects to plant allelochemicals [34], [35]. The array of substrates of the respective enzymes has been shown to be correlated with the feeding habits of the insects, *i.e.*, a broad substrate specificity in generalists that feed on a wide diversity of host plants containing a broad spectrum of allelochemicals, and a narrow substrate specificity in specialist insects that feed on only one or a few plant species [36]. The same has been shown for FMOs involved in PA *N*-oxygenation of a specialist and a generalist arctiid species [17]. Concretely, the respective enzyme of the specialist *T. jacobaeae* is highly specific for PAs of the toxic senecionine type, the predominant PA type of its almost exclusive food plant *Jacobaea vulgaris* (syn. *Senecio jacobaea*). In contrast, the *N*-oxygenase of the generalist *G. geneura* (Syn.: *G. incorrupta*) accepts a wider range of substrates, including the non-toxic 1,2-saturated PA phalaenopsine and the tropane alkaloid atropine [17]. Most interestingly, the substrate specificity of the PA *N*-oxygenase of *G. geneura* is almost identical to that and of the PNO of *Z. variegatus* that also accepts atropine as a substrate. We postulate that the almost identical substrate specificity of the PA *N*-oxygenases of the two unrelated insects is the result of convergent evolution under identical selection pressure of generalist feeding. The wide substrate specificity of the PA *N*-oxygenase of *Z. variegatus* correlates with the extremely polyphagous behavior of this grasshopper, which is ranked as one of the most important economically pests of agriculture in west and central Africa [37], [38].

Of note, the *N*-oxygenation of PAs in specialized insects is not a detoxification mechanism that converts xenobiotics into more polar metabolites for efficient excretion from the body. Instead, this enzymatic conversion allows the sequestration of these plant toxins in a deactivated, metabolically safe form [5]. The recruitment of an enzyme that catalyzes PA *N*-oxygenation can be regarded as a “key innovation” during the insect's adaptation to PA-containing food plants [17]. In arctiids, this enzyme was the prerequisite for the evolution of several further, highly specific adaptations, such as the positive feeding response triggered by traces of PAs sensed by specialized taste receptors, the ability to transfer the alkaloids from the larvae to the adult stage, and the insect's behavior that ensures optimal egg protection by PAs of both parents governed by PA-derived pheromones [5], [39].

The high degree of sequence identity of the PA *N*-oxygenase to two further FMOs identified from *Z. variegatus*, and the fact that the latter two enzymes are also able to catalyze the *N*-oxygenation of alkaloids, albeit with a much lower specific activity, indicate the close relationship of these sequences. The data suggest a duplication of an ancestor gene encoding an FMO of unknown function that already possessed the ability to *N*-oxygenize PAs, most probably as a side activity. One of the gene copies was recruited and optimized for *N*-oxygenation of plant-derived PAs. The finding that PAs are strong phagostimulants for *Zonocerus* [40] suggests a central role of PAs in the insect's ecology. Further research has to show whether this is also the case for the *N*-oxygenation of further plant-derived toxins, such as atropine or nicotine, that are accepted as a substrate by the PNO of *Z. variegatus*.

## Supporting Information

Figure S1 **Amino acid alignment of flavin-dependent monooxygenases of various insect species.** Only the central part of the alignment that was used for the estimation of the phylogenies is shown, spanning the region from amino acid position 5 to 402 with respect to *Zv*PNO. The sequence motifs for binding of FAD and NADPH and the FMO-identifying sequence are boxed. The accession numbers of the three FMO sequences of *Zonocerus variegatus* and of all sequences taken from the databases are given at the end of the alignment.
(PDF)

Figure S2 **PNO activity in three buffer systems with pH variation.** Enzyme activities have been determined with senecionine as substrate at 37°C.
(PDF)

Table S1 **Sequences of primers used for the identification and cloning of cDNAs of flavin-dependent monooxygenases of** ***Zonocerus variegatus*** **.** Recognition sites of restriction endonucleases used for cloning are underlined.
(DOC)
