# Supplementary material for: Independent Recruitment of a Flavin-Dependent Monooxygenase for Safe Accumulation of Sequestered Pyrrolizidine Alkaloids in Grasshoppers and Moths
Source: PLoS One. 2012 Feb 20;7(2):e31796. doi: 10.1371/journal.pone.0031796 (PMC3282741; doi:10.1371/journal.pone.0031796)
Supplement: Figure S1 — Amino acid alignment of flavin-dependent monooxygenases of various insect species. Only the central part of the alignment that was used for the estimation of the phylogenies is shown, spanning the region from amino acid position 5 to 402 with respect to ZvPNO. The sequence motifs for binding of FAD and NADPH and the FMO-identifying sequence are boxed. The accession numbers of the three FMO sequences of Zonocerus variegatus and of all sequences taken from the databases are given at the end of the alignment. (PDF) [file pone.0031796.s001.pdf]

```
                 FAD-site 10        20        30        40        50        60        70        80        90       100
             ...       |....|....|....|....|....|....|....|....|....|....|....|....|....|....|....|....|....|....|
TcFMOa       AIIGAGKAGLCAGKHCLKENISFD--IFEQTGNLGGTWFYTDLVGHDENGAPIHTSMYKGLRTNLPNELMTFEDFPYPKQIR-SYLLQEEVLDY------
TcFMOb       AIIGAGAAGLCAGRHCLRENIAFD--IFEQTGNLGGTWNYTDLVGCDENGVPIHSSMYKGLRTNLPKELMAFEDFPYPKQNR-SYLLQDEVLDYVRSYSD
AgFMOa       CVIGAGTAGLCAARHALQAGGIVT--VFEMGKQLGGTWVFNEATGKNEYGIDVHSSMYKGLKTNLPKEIMGYPDFPIPEQ-ESSYIPAEDMLTFFQQFAE
AgFMOb       CIIGAGMAGLAAARRVLEIGAQVT--IFERMDQLGGTWIYTDEVGVDRYGLPVHTSMYRGLRTNLPKEVMGYPDFPIPAQ-RDSYIVSDDILSFLRLYAD
DmFMO3006    CIIGAGTAGLCCARHSIANGFETT--VFELSDRIGGTWVYNEATGAVN-GIDVHSSMYKNLRTNLPKEVMGFPDFEIGAN-EASYVRSDEICDFLNQYAN
DmFMO3174    CVIGAGTAGLCALKNSLEAGLDAV--AYERGTEIGGTWIFSEEMPKDEYD-EVHSSMYEGLRTNLPKEVMGYPDYSYPDDITESFITSNQVLEFLRSYAE
TcFMOc       AIVGAGAAGLASARHVSAQGIDCD--VLEMGPELGGTWVYTDNVGSDQYGFPVYSAMYKGLRTNLPKEVMGYPDFPIPEQ-NKSYLTQAEILDFLNLYAD
TcFMOd       AIIGAGAAGLASARHVSAQGIECE--VIEMGSEVGGTWVYTDEVGTDRFGYPVHTAMYKGLRANLPKEIMGFPDFPIPEP-NGSYLDQATILRFLNLYAE
BmFMO3       CVIGAGAAGVCAARHLLVEPCVDHVDIFEQASQLGGTWVYTEKVGYDDFGLPIHSSMYKSLRTNLPKEIMGFPDFPVPES-EKSYLPAKEMLSFLQLYAD
HaFMO3       GLIGAGAAGLCAARHLLEEPCVSHVDILEQAPQLGGTWVYTENVGYDDFGLPIHTSMYKSLRTNLPKEIMGFPDFPVPES-EQSYLPAKDMLAFLKLYAD
AcFMO        CIIGAGYSGLATARHMIDYGLNLT--VFEVPNYIGGTWRYTPRVGTDEYGAPLFTSAYKDLRTNSFYPTMELPDYPFPAGPVSSFLSGPCIYKYLEGYTK
TjFMO        CIIGAGYSGLATARHMIDYGLNLT--VFEASSYIGGTWKYTPRVGTDENGAPLFTSAYKNLRTNSFYQTMEFPDYPFPQGS-SSYLSGPCIYKYLQGYTK
AcPNO        CIIGSGYSGLAAARYMQNYGLNYT--VFEATRNIGGTWRYDPRVGTDEDGLPIFSSQYKNLRINSPYKIMEFHDYSFPEG-TRSFISGGCFYKYMKSFVR
GgPNO        CIIGSGYSGLAAAKYMQDYGLNYT--VFEATRNIGGTWRYDPRVGTDEDGLPIFSSQYKYLRTNSPYKIMEFHDYPFPEG-TRSFITGGCFYKYMKSFVR
TjSNO        CIIGAGYSGLATARYLQDYGLNYT--IFEATPNIGGTWRYDPRVGTDEDGIPIYSSNYKNLRVNSPVDLMTYHGYEFQEG-TRSFISGNCFYKYMKSFVR
BmFMO1       CIIGAGYSGLGAARYMKQYHVNFT--VFEATRNFGGTWHFDPHVGTDEDGLPVFSSMYNDLRTNTPRQTMEYYDFPFPEG-TPSYPSATCFLDYLKSFVK
HaFMO1       CIIGAGYSGLGTARYMREYGLNFT--VFEASRHIGGTWRFDPHVGTDEDGLPLFTSMYKNLRTNTPRQTMEYAGFPFPEG-TPSYPTGPCFYKYLQHFAK
BmFMO2       CVIGAGIAGLSSARYLKEEGIDFV--VFEATKYIGGTWRYDPRVGTDENGLPLHTSMYKHLHTNLPKPTMELRGFPLPDG-IPSFPSWKIYYDYLKDYAK
HaFMO2       CVIGAGIAGISSARYLKEEGIDFT--VFESTRYIGGTWRYDTHVGTDENGQPLHTSMYKYLRTNLPKAPMEMRGFPLPDY-LPSYPTGRDFYHYLEECVD
ZvFMOa       AVLGAGPCGLATARHLKHAGFEVT--VFERSRHVGGTWNYTDETWMSEDGRPIYTSLYQNLVVNLPKEIMAFPDFPF-HHVDDSYVSSKEVLKYFNNFCD
ZvFMOc       AVLGAGPSGLVAARYLKDAGFEVT--VYERLHHVGGTWNYTDETWMAEDGRPIYSSMYQNLLVNLPKEIMAFPDFPF-HDIEESYVPSKEIWKYYNNFCD
ZvPNO        AVLGAGPSGLTAARYLKQAGFEVM--VFERYHHVGGTWNYTDETWMSEDGRPVYSSMYQNLFVNLPKELMAFPDFPF-HDIEGSYVPSKEVLKYFDNFTD
```

```
                    110       120       130       140       150       160   FMO identifying sequence  190  NADP-site
             ....|....|....|....|....|....|....|....|....|....|....|....|...           |....|....|...       |
TcFMOa       ---------YFKRVIWIER----QNFLWSVHYEDVKNKQKEMEHYDAVIICNGHYSDPFIPDIPGIESFSGKVKHSHDYRTPEPYANKKVLILGSGPSGL
TcFMOb       KFHINPHIKYFKRVIRIER----QNFLWSVHYEDVKNKQKDMEHYDAVIICNGHYSDPFIPDVPGIESFSGRVKHSHDYRTPEPYANKKVLILGSGPSGL
AgFMOa       SYGILEHIRFSHYVVRVKPTI--DEKGWEVIVRDCPNDQLLTLTFDYVLVCNGHYHTPNLPKYPGMSVFRGKQMHSHDYRSNEPFEGETVLVIGAGPSGM
AgFMOb       RYHIKDCIKFEHHVVQVHPT---DGERWIVEVENLADHQKEQHVFDYLFICNGHYHTPNVPTVNGSEIFQGQQLHSHDYRCTEHYKDKAVLVIGAGPSGM
DmFMO3006    HFDLKKHIKFDSYVIRVLQ----RKTKWQVLFKDLVTNKIEFQYFDKVLVANGHYHTPNYSQIPNMERFKGQFLHSHDFRSREVFEGKSVLVIGAGPSGM
DmFMO3174    HFKLKAHIKLQHEVIRVRP----RLDDWEVYVWDHSTDTCDPVYYDFVYVCNGHYTEPDLPEVEGLDLFEGNKMHSHLYRKADKFKDARVLIIGAGPSGM
TcFMOc       HFHIRQHIRFNRMVVEIRP----LGDKWQIKSIHKPTKEEIVDIYDAVMICNGHYNDPIIPKIPGQEKFKGEIAHSHQYRSPERFKNQNVLVIGAGPSGL
TcFMOd       HFNLKPLIKFNHIVTEVRP----NADKWSIKAKNKITKTEFASIYDVVMICTGHYNTPISPSLSGQEKFKGHVMHSHQYRSNKPFQNQRVLVIGAGPSGL
BmFMO3       KHQVTDRINFNHHVNLVIPKAGPSGELWDVSFKNLLNGESETREYDYVFVCNGHYNTPFIPNIPGLKEFQGDVMHSHDYRVPEIFSGKRVLVVGAGPSGM
HaFMO3       KHGVTEKIKFSHHVQLVIPKQGPSGELWDVSYKNLLNGHSETREYDYVFVCNGHYNTPFIPQIPGLKEFQGDVMHSHDYRVPDIFTDKRVLVIGAGPSGM
AcFMO        QFNLKKYIQFRSLVTSVEK----VGDNWKVTYMKTDTKQNVSEECSFVVVANGEYTAPHVPYFAKQEDFKGKMLHSHDYRDSEDYRGLRVLVVGAGPSAF
TjFMO        QFNLEKHIKFQSLVTSVER----VGDMWNVTYMKTDTKENVSEECGFVVVANGEYIAPHIPYFAKQEDFQGKMPHSHDYRDSEDYRGLRVLVVGAGPSAF
AcPNO        HFGLMDNIQVQSLITWVEW----TGYSWNLTYMKTDTRQNYTEECGFVVVATGEYSTPKIPHIKGQELYKGKTMHSHDYKDPEDFRGQRVMLIGAGPSGL
GgPNO        HFKLMDNIQVQSLVTWVEW----TGDNWKVTYMKTDTRKNYTEECDFVVVATGEYSTPKIPHIEGQELYTGKTMHSHDYKDPEDFRGQRVMLIGAGPSGL
TjSNO        HFGLMENIQVRSLVTWVQR----TEDKWNLTYMKTDTRKNYTEECDFVVVASGEFSTPKIPHIKGQEEYKGKTMHSHDYKEAESFRGQRVLVIGAGPSGL
BmFMO1       HFDLLSHIQLRSLVTSVKW----AGNHWNLTYTKTDTKENVTETCDFIVVANGPYNTPVWPKYDGIDTFEGSMIHSHDYKDRKAYKNRKVLIVGAGASGL
HaFMO1       HFELMNNIQLQSYVNLVKW----AKDHWEVTYTKTDTKEQLTEVCDFIVVASGEFSSPVIPNIDRLEMFKGKVIHSHDYKDAEEFRNRRVLLVGAGASGL
BmFMO2       HFDIEKYIQFRHNVTLVRR----EQNVWKVTHEHVITGEVFEENYDYVIVGNGHFSTPNMPNIRGEKLFKGTIIHSHDYRVPDVYKDRRVLVVGAGPSGM
HaFMO2       RLDIKKYIKFLHAVVSVRR----INEVWKVKYEHVVTKETFEEDFDYIIVGNGHFSKPSYPNIPSEDLFTGRIIHSHDYKAPEPFTNRRVLVVGAGPSGM
ZvFMOa       AFDLRKLVKFQHHVENVRP----CDSGWLVTVTDLTTMMEQSFEFDAVAVCTGQCWCPLYPNVEGRTIFRGRQIHAHEFRCPDSFRNRRVLVIGAGPSGH
ZvFMOc       SFDLRKLIKFHHHVENVRP----CDSGWLVTVTDLTNMVEHSSEFDAVVVCTGQCWCPLYPNVEGSNNFRGRQTHAHTYRNPDSFRNRRVLVVGAGPSGH
ZvPNO        AFDLRKLIKLQHHVENVRP----CESGWLVTVTDLTTMVEHSFEFDAVVVCTGQTWCPLYPDVEGRSFFRGRLTHAHEFRSPEPFRNKRVLIVGAGPSGH
```

```
                           210       220       230       240       250       260       270       280       290       300
                  ....|....|....|....|....|....|....|....|....|....|....|....|....|....|....|....|....|....|....|....|
TcFMOa            EISQQISNVATKVFISHRSKDA---LP---VSDALYQKCLVKEFV-ENRAIFEDGTSEEIDDVVFCTGYNYNFPFLSK-RCGVKITNNYVHPLYKQIISI
TcFMOb            DISQQISNVATKVFLSHRSKDP---LP---VPDILHQKCLIKEFV-ENKAIFEDGTSEEIDDVVFCTGYNYNFPFLST-NCGVKITDNYVHPLYKQIISI
AgFMOa            DMAYEISKKAIRVTLSHHLKD----KPQTVFPSNVTLKPDVTRLT-ETGVEYADGTSEDFSVICYSTGYKYTFPFLSV-DCGITVEENYVQPLYKHCINI
AgFMOb            DIALELAKTARRVTISHHME-----RLTFPFPSNLSQQSDVSMLT-ETGAKFTNGSEESFDVVLYCTGFRYNFPFLGA-DCGIEVQDNHVQPLYKHCINI
DmFMO3006         DLSNIISRTADRVTISHHLTD----IGQHSFFENVQQKPDVRELD-EKGAFFVDGSYQEFDTVFFCTGYKYAFPFLTV-DSGIYVEDNYVQELYKQCINI
DmFMO3174         DITNHVRLAAKQVFLSHHLST----TPNTAFMGNVTQKPDVKRFT-KDGAVFTDGSTESFDHVMFCTGYKYTFPCLST-DVGVQVIDNFVQPLWKHCINI
TcFMOc            DLALHISSVAKQVVLSHHTKE----AVNTEYPCNVSKKPDVSAIKGEEEVEFVDGSCCRFDTIIYCTGYRYSFPFLHE-SCGVTVDDNHIQPLYKHMIHI
TcFMOd            DVAFQVAEIAQQVVLSYDMTKK---EVKGEYPSNLVKKPQVLRVKDKEHVEFVDGSCCSFDTIIYCTGYRYNFPFLHH-DCGVSVGDFHVRPLYKHLIHI
BmFMO3            DIALEVTNVAHKVILSHHL-KE---QPRTVFPDNLTQKPDVKRLDGKK-VHFADESEDEVDVVFLCTGYLYNFPFLHE-SCNISVEDNCVEPLYKHLVNI
HaFMO3            DIALELTSVSKKVILSHHL-KD---QPRTVFPENLEQKPDVERLDGHK-ACFLDGTEDEVDVVFLCTGYLYNFPFLHE-SCGIVVEDNCVEPLYKHVVNM
AcFMO             DLAAHLINVTSMFIHSHHLDAKI-----QKVYGNYKRKPDIKHFT-PTGAVFVDDTTEQFDVAILCTGYSYSFPFLNYQSSGVTSSAKYIMPLYNQLINI
TjFMO             DLATHLINVTSMFIHSHHLDAKL-----PEVYGNYKRKPDIKHFT-PTGAVFVDGSTEEFDVAILCTGYKYSFPFLNYKSSGVAWTDKYVMPLYNQLINI
AcPNO             DLAVQLSNVTSKLVHSQHIIKSFKIYNQPDFPGNYISKPNVKYFT-PNGAVFEDDTSEEFDIIIYCTGFYYNHPFLSTQSSGVTTTENYVMPLYQAVVNI
GgPNO             DLAVQLSNVTSKLVHSHHIIKSFKIYNQPDFPGNYISKPNVKYFT-STGAVFEDGTTEDFDIVIYCTGFYYNHPFLSTQSSGVTTTENYVMPLYQAVVNI
TjSNO             DVVMQLSNITSKLVHSQHILKSWHIFNQPDFPGNFISKPNVKHFT-ANGAVFEDDTVEEFDMVIYCTGFYYNHPFLSTLSSGITATENYVMPLYQQVVNI
BmFMO1            DLAIQLSNVTAKLVHSHHLVYN-----EPKFFDGYVKKPDIMAFT-PKGVIFRDESFEELDDVIFCTGYDFNHPFLD-ESCGVTSTAKFVLPLHKQLVNI
HaFMO1            DLAMQLSNVTSQLFHSHHLNYN----QPDFSK-TYVKKPDIDSFT-PTGAFFVDGSTEEFDDVIFCTGYNYNHPFLD-SSSGVTASRKFVLPLYQQTVNI
BmFMO2            DIGLDVAECSKSLLHSHHS-KVN--FRTPFPP-HYVRKPDVKEFN-ETGVIFVDGTYEEIDDVIYCTGFQYDYPFLD-KTCGLDIDPHSVVPLYKYMVNI
HaFMO2            DIGLEVADVASALIHSHHS-KI--NWTTPFPP-HYHKKPDIKEFN-ETGVIFEDGSFEEIDDVIYCTGFYYDFPFLD-ESSGLTMEPKSVVPLYRYTVNI
ZvFMOa            DLALNISYVAKQVFISRRELK----TVEGLFPDNVTEKPLLTSLS-EYTAHFSDGTSTDIDDIIYCTGYRFRFPFLSP-ECGVIVDEKRVHPLYLHVLNI
ZvFMOc            ELALIISYVAKQVFLSRRELK----IVEGLFPDNVTEKPLLTSLT-EYTAYFSDGSSIDIDDILYCTGYRFRFPFLSP-ECGIIADEKRVHPLYMHVLNI
ZvPNO             DMALHISYVSKEVFLSRKELK----PVEGLFPDNVTEKPLLTSLS-EYTAHFSDGTSTDVDEILYCTGYRYRFPFLSP-ECGVTVDEKYVYPLYLHMLNI

                           310       320       330       340       350       360       370       380       390       400
                  ....|....|....|....|....|....|....|....|....|....|....|....|....|....|....|....|....|....|....|
TcFMOa            ENPTLAFIGIPFKACPCPLFDIQVRFVLASLTGHFKLPKKDVMLKELVEE--EKRKPGPP-SSQYHQLGGAQGSYFDNLAETAKIRKIPPVIQKLYLRVI
TcFMOb            ENPTLAFLGIPFKVCPFPLFDIQVRFFLATLTGHFKLPKKEDMLQELVEE--EKRKSGLP-RPKYHELGKAQGSYFNDLSETAKIKMVPQVVQKLYVRVM
AgFMOa            RYPTMAFIGLPFYVCAAQMMDLQARFCLKFFSGAKTLPTQEEMSADTAAEMEERWKRGLK-KRQAHMMGPVEDRYYDDLAQTAEIDPIKPVIKKLHKISA
AgFMOb            NHPTMAFIGLPFYVCAAQMMDLQVRFCLAYLTGRQRLPPAHEMLDDAAQEFEDRLQRGYK-KRHAHMMGPEQGRYYDDLAKTAQIETIPLVMTKLHNESS
DmFMO3006         RNPSMALIGLPFYVCAAQMMDIQARFIMSYYNGSNELPSTEDMLKDTRDRMGKLWAEGLR-KRHAHMLGPKQIDYFTDLSQTAGVKNIKPVMTKLHNESS
DmFMO3174         NHPTMAFVGLPFNVIPTHIFDMQVRFTLKFFTGQRKFPSREQMIADLEQEIGERWGCGVRNQKKAHQMGERQFVYYNELASIAGIENIKPVIHKLMKDCG
TcFMOc            ERPTMCFIGIPFNVCAFQMFDLQARFFCQYLNGSMSLPSCNMMRMDTEKDMQNRWAKGYT-KRQAHLMGPDQQGYYEDLAAAANTTPIAPVIVKLRDESV
TcFMOd            EKPTMCFIGIPYYVGAFQMFDIQARFYCQYLNGSMSLPTKEMMYKDTEEDVVKRKNKGYS-EKQMHLLGHDQQTYFEELASTAKITPILPVICKIWSDSD
BmFMO3            HHPTMCFIGVPYYVCAFSMFDLQVRYYIRSINGTFSLPSTEEMIAHWEEEKKDRASRGYT-KRQAHMMGPDQASYYMSLSEESETVPLPPVFTSIHNDSS
HaFMO3            NHPSMCFIGVPYYVCAFSMFDLQVRYYVRSMNGTFSLPSTEEMAQHWEEEKRDRAARGYT-KRQAHMMGPDQEKYYASLATEAKTKTLPSVMTKIRDESS
AcFMO             NHPTMTFVG-TGKYSIGLVRDRQGHYSAQLAAGLVKLPSKDEMFQEWFDHA---KHQTPK---EINLIGYENTANYMDTLLNGTDIPGVPLVFTTILRNH
TjFMO             NYPTMTFVG-TGKYSIGLVRDRQGHYSAQLAAGLVKLPSQDEMFKQWFDYT---KHQTAK---EINLIGYSNTESYMETLLNGTDIPRPPPVFTTILRNH
AcPNO             NQPTMTFIG-ICKPGFAKILDLQAQYSAALAAGQFKLPTKDTMLRHWLEHVHMLKESQYK-ITDVNSVGINIDKYFEALHKEAGVPLLPPVYTSMFTFSG
GgPNO             NQPTMTFVG-ICKPFYAKILDIQAQYSAALAAKKFELPSKDSMLRHWLEHVYALRQSQFK-VTNINAIGSNIDQYFEALHKEAHVPLVPPVYTSMFTFSG
TjSNO             NQPTMTFVG-ICKPFFAKLLDQQAHYSAKLAAGHFKLPSQDKMLRHWLEHVQMLREAQFK-ITDVNSVGPNVDEYFKALHKEAGVPLLPPVYASVFVFSG
BmFMO1            KHPSMVFLG-IAKKIITRVMDAQAEYAALLASGKLKLPSQEEMLNSWLKHISSLQVKGMK-IIDLNVVGSEMDQYFGNLTEEAGVVRAPPVLTAIRDFNG
HaFMO1            KHPSMTFVG-VSKKVINRVMDAQGQYAAALASGKFQLPSQEEMLRNWLEHVYAQQNKGMR-IVDVNVI-SDMDSYFGNLTLEAEITPAPPVLTKIARFNG
BmFMO2            RQPSMVILGLVVRACLVVALDAQARYATALIKGNFTLPSEAEMMDEWQRRADAIRSKGLR-MSHIHTLAEKEDEYYAELSEQSGIERVPPVMFKIRAMDI
HaFMO2            NQPSMFIMGAFIRACLVVALDAQARYATAYIKGNFSLPTRDEMMLEWQKRMDTIRSKGLP-TSYIHILGEKEDEFYAELTRESGIERVPPVMFKIRTMDT
ZvFMOa            NKPTMGFVGVPHDACFSILFDLQAQWFTAVLAGRCTLPDAETMRKEEEEELERQLAAGF----RPHFMYNRQWKYFKQLEDMAGAKPMPLYYMKMFDDLA
ZvFMOc            NNPTMGFIGVPPAACFSVLFDLQAQLFTAVLTGRCNLPDAETMRKEEEEELERQLAAGF----QPHFMANRQWKYFKQLEDMAGAKPVPLYYMKMFDDVV
ZvPNO             NKPTMLFIGVSYNACYSIMFDLQAQWVTAVLAGRCTLPDAETMRKEEAEYMEKQRAEAV----HPHVLMNHQWEYFKKLEEMSGAKTMPPVYMKMFDDVA
```

	410<br>....|....|....|	Organism	Acc. No.
TcFMOa	KHRKLRPAFNKLLAW	Tribolium castaneum	XP_001810900
TcFMOb	MNRNRNNCFEIIDDD	Tribolium castaneum	XP_969414
AgFMOa	MRYSEDLVNFRNDKF	Anopheles gambiae	XP_311551
AgFMOb	QRFVDDLIHFREDVF	Anopheles gambiae	XP_311550
DmFMO3006	KCFNENLLHFREDNF	Drosophila melanogaster	NP_611859
DmFMO3174	KKYIFELDTYRSNKY	Drosophila melanogaster	NP_610217
TcFMOc	KRLYDDLLNFREDRY	Tribolium castaneum	XP_973683
TcFMOd	NSFYKDLKNFRKYKY	Tribolium castaneum	XP_969340
BmFMO3	QKFLDNLTSYRDDVY	Bombyx mori	ADH16749
HaFMO3	IRFLHNLKHYRQDVY	Helicoverpa armigera	ADH16754
AcFMO	IDLWYTEFLTFRNYQ	Arctia caja	CBI83750
TjFMO	IDIWYTEFLTFRNYQ	Tyria jacobaeae	CBI83753
AcPNO	KTLLEDLLHYRDYDY	Arctia caja	CBI83746
GgPNO	KTLLEDLLNYREYDY	Grammia geneura	CBI83748
TjSNO	KTLLEDLQNYREYDY	Tyria jacobaeae	CAD12369
BmFMO1	VNRLDDLLNYREYDY	Bombyx mori	ADH16746
HaFMO1	KNRLEDLLNYRDYDY	Helicoverpa armigera	ADH16751
BmFMO2	EAKLENLYTYRHYVY	Bombyx mori	ADH16748
HaFMO2	EAKIENLYTYRNYAY	Helicoverpa armigera	ADH16752
ZvFMOa	PGLTKDLQHYRKNKY	Zonocerus variegatus	CBX26643.1
ZvFMOc	FDWAKDLQHFRRNNY	Zonocerus variegatus	CBX26644.1
ZvPNO	SDLVKDLQNFRKNNY	Zonocerus variegatus	CBX26645.1
