# Supplementary figures and images for: Independent Recruitment of a Flavin-Dependent Monooxygenase for Safe Accumulation of Sequestered Pyrrolizidine Alkaloids in Grasshoppers and Moths
Source: PLoS One. 2012 Feb 20;7(2):e31796. doi: 10.1371/journal.pone.0031796 (PMC3282741; doi:10.1371/journal.pone.0031796)

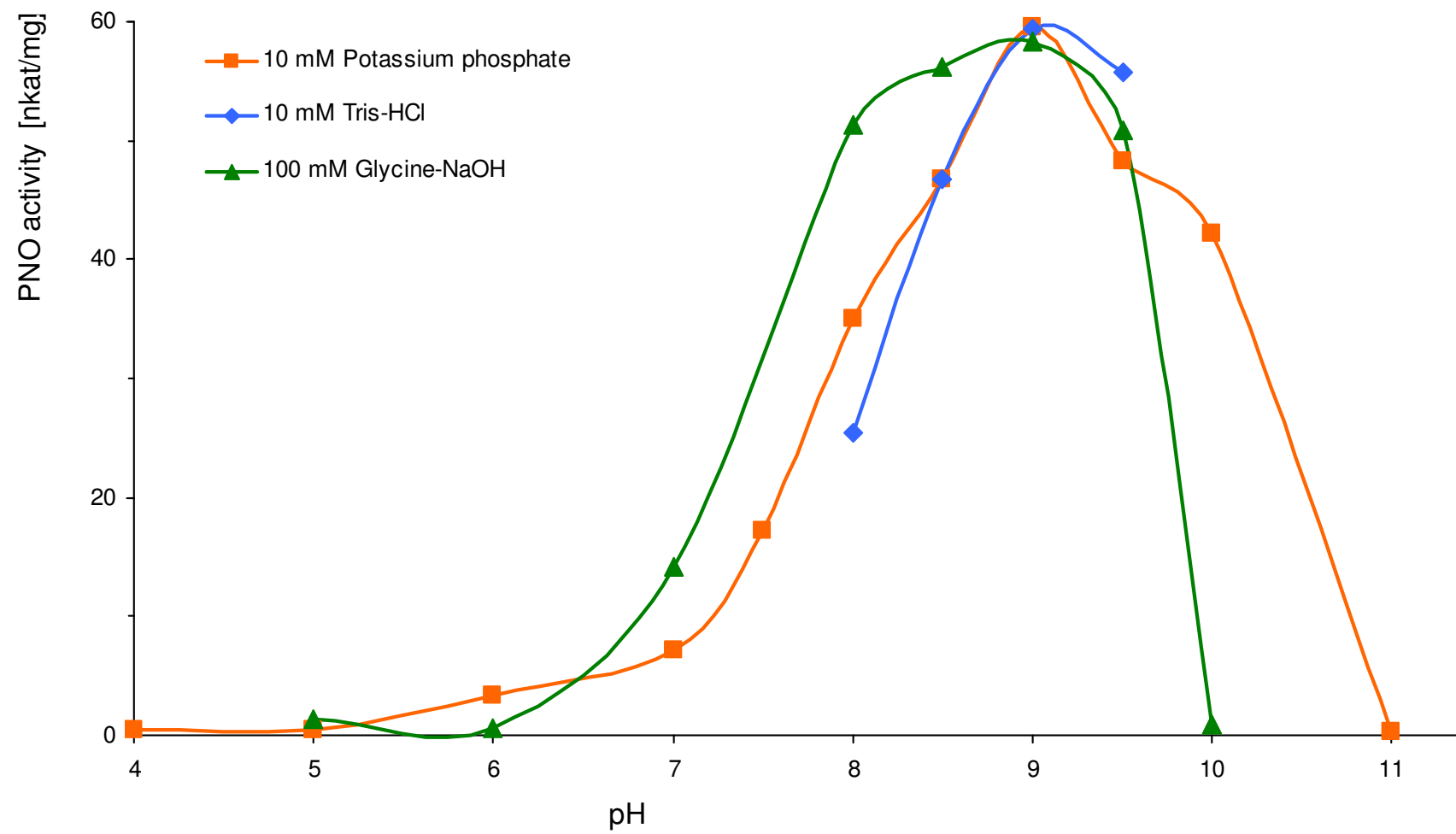

Supplement: Figure S2 — PNO activity in three buffer systems with pH variation. Enzyme activities have been determined with senecionine as substrate at 37°C. (PDF) [file pone.0031796.s002.pdf]
