# Supplementary material for: Independent Recruitment of a Flavin-Dependent Monooxygenase for Safe Accumulation of Sequestered Pyrrolizidine Alkaloids in Grasshoppers and Moths
Source: PLoS One. 2012 Feb 20;7(2):e31796. doi: 10.1371/journal.pone.0031796 (PMC3282741; doi:10.1371/journal.pone.0031796)
Supplement: Table S1 — Sequences of primers used for the identification and cloning of cDNAs of flavin-dependent monooxygenases of Zonocerus variegatus . Recognition sites of restriction endonucleases used for cloning are underlined. (DOC) [file pone.0031796.s003.doc]

**Table S1. Sequences of primers used for the identification and cloning of cDNAs of flavin-dependent monooxygenases of *Zonocerus variegatus*.** Recognition sites of restriction endonucleases used for cloning are underlined.

| No. | Primer Sequence | Properties |
| --- | --- | --- |
| P01 | 5’-dGTC GAC TCG AGA ATT C(T)_17_-3’ | oligo(dT) primer |
| P02 | 5’-dGGN GGN ACI TGG MGN TAY-3’ | degenerate primer |
| P03 | 5’-dGGN CCN GCI CCD ATN AC-3’ | degenerate primer |
| P04 | 5’-dAAA AGA GAT TAT GGC TTT CCC CGA TTT T-3’ | 3’RACE ZvFMOa and ZvFMOc |
| P05 | 5’-dGGT ACG TCC CTC CAC ATT T-3’ | 5’RACE ZvFMOa |
| P06 | 5’-dAGT ACA TAC AGC GAC TGC GTC AAA TT-3’ | 5’RACE ZvFMOa |
| P07 | 5’-dAAA CTT CAC CAG CTT CCT AAG GTC AAA-3’ | 5’RACE ZvFMOa |
| P08 | 5’-dAGA ATG TCA TCG ATG TCA ATG-3’ | 5’RACE ZvFMOc |
| P09 | 5’-dTCT GCC GTC CAC GGA AAT TGT TAC TC-3’ | 5’RACE ZvFMOc |
| P10 | 5’-dCTA CGT GAT GAT GAA ACT TGA TCA GTT TT-3’ | 5’RACE ZvFMOc |
| P11 | 5’-dTAT ACA TAT GCG TCA GGT CGC TGT ATT GGG AGC A-3’ | Expression ZvFMOa |
| P12 | 5’-dTAT CTC GAG TAT CTT TTC ATA ATT TTC GTT GTC GAC AAT-3’ | Expression ZvFMOa |
| P13 | 5’-dTAT ACA TAT GCG TCG GGT GGC TGT ATT GGG AGC-3’ | Expression ZvFMOc |
| P14 | 5’-dTAT CTC GAG GTA TAT CTT TTT ATA ATT TTC GTT GTC GAT T-3’ | Expression ZvFMOc |
| P15 | 5’-dGGT CGT TGT ATG TAC TGG GCA AAC-3’ | 3’RACE ZvPNO |
| P16 | 5’-dATG TGG TGT TAC TGT AGA CGA GAA GTA-3’ | 3’RACE ZvPNO |
| P17 | 5’-dTAG AAT TTC ATC GAC GTC AGT A-3’ | 5’RACE ZvPNO |
| P18 | 5’-dAAT CAG AAC GCG TTT ATT CCT AAA AGG T-3’ | 5’RACE ZvPNO |
| P19 | 5’-dAGC TTC CTA AGG TCA AAG GCA TCA GT-3’ | 5’RACE ZvPNO |
| P20 | 5’-dTAT AGG TCT CAC ATG CGT CGG GTG GCT GTA TTG GGA G-3’ | Expression ZvPNO |
| P21 | 5’-dTAT AGC GGC CGC GTA TAT CTT TTT ATA ATT TTC GTT GTC GAT A-3’ | Expression ZvPNO |
